# Biomedical heterogeneous data categorization and schema mapping toward data integration

**DOI:** 10.3389/fdata.2023.1173038

**Published:** 2023-04-17

**Authors:** Priya Deshpande, Alexander Rasin, Roselyne Tchoua, Jacob Furst, Daniela Raicu, Michiel Schinkel, Hari Trivedi, Sameer Antani

**Affiliations:** ^1^Marquette University, Milwaukee, WI, United States; ^2^DePaul University, Chicago, IL, United States; ^3^Center for Experimental and Molecular Medicine (CEMM), University of Amsterdam, Amsterdam, Netherlands; ^4^Emory University, Atlanta, GA, United States; ^5^National Library of Medicine, National Institutes of Health, Bethesda, MD, United States

**Keywords:** data categorization, data integration, datasets, heterogeneous data, schema mapping, semantic similarity, unstructured data

## Abstract

Data integration is a well-motivated problem in the clinical data science domain. Availability of patient data, reference clinical cases, and datasets for research have the potential to advance the healthcare industry. However, the unstructured (text, audio, or video data) and heterogeneous nature of the data, the variety of data standards and formats, and patient privacy constraint make data interoperability and integration a challenge. The clinical text is further categorized into different semantic groups and may be stored in different files and formats. Even the same organization may store cases in different data structures, making data integration more challenging. With such inherent complexity, domain experts and domain knowledge are often necessary to perform data integration. However, expert human labor is time and cost prohibitive. To overcome the variability in the structure, format, and content of the different data sources, we map the text into common categories and compute similarity within those. In this paper, we present a method to categorize and merge clinical data by considering the underlying semantics behind the cases and use reference information about the cases to perform data integration. Evaluation shows that we were able to merge 88% of clinical data from five different sources.

## 1. Introduction

In the healthcare domain, data integration plays an important role in data science applications for improving patient care and aiding clinical research. However, it is hampered by the heterogeneous and unstructured nature of medical data. Our ability to merge data across hospitals and research institutions remains limited due to the lack of annotations or other descriptive category information, because this information is available, it can be further used toward knowing data elements properties for integration purposes. There is a significant need to design and develop a data integration system that can enable the reuse of multi-institutional data by researchers and clinical practitioners (Meystre et al., 2017). In this work, we describe a methodology for data integration that enables the merging of data from heterogeneous biomedical sources based on the semantics of their data elements. We map the biomedical data elements (clinical cases) from different, but related, datasets into categories and merge sub-categories to design an integrated database schema.

We perform the categorization by classifying sub-categories from medical data sources, extracting semantics of their data elements, computing their semantic similarity, and integrating those that meet a predetermined similarity confidence threshold. We set this confidence threshold based on empirical analysis and evaluation of different cases.

**Figure 1:**
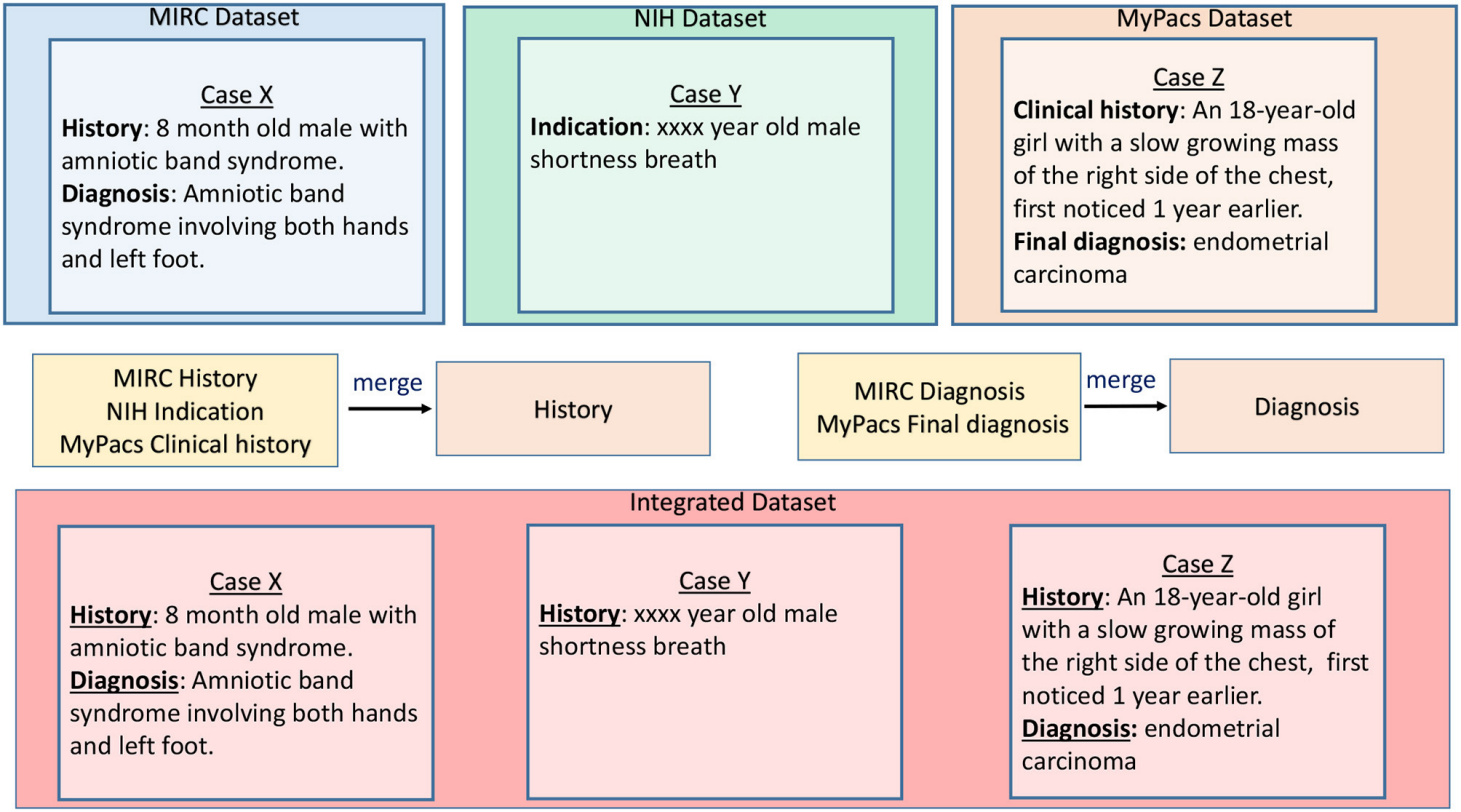
Super-category combination sample example.

To integrate data sources, we cluster sub-categories and identify the super-category combinations that can be merged. Once we merge the sub-categories based on their semantic similarity, we identify different attributes for schema design based on the contents of merged data elements and design an appropriate database schema. For example, sub-categories with findings, observation, and diagnosis would be treated as one category (with similar representation pattern), while another category could integrate cases that include patient clinical history, e.g., discussion, history, or comments. As illustrated in Figure 1, we find super-category combinations that we can merge to find the super category (final category) for the data integration purposes. Using our data categorization and merging technique, we were able to merge 88% of sub-categories—reducing original sub-categories to 35% (total 82 sub-categories from original data sources reduced to 29 merged categories) from five public medical datasets [EURORAD (Neutorgasse, 2017), MyPacs (Group, 2017), MIRC RSNA,^1^ NIH clinical reports NIH,^2^ and NIH x-ray NIH^3^].

### 1.1. Contributions

#### 1.1.1. In this paper we present

Algorithms that extract semantic information from unstructured data and find attributes for developing a schema for integrated data repository.

A methodology for data integration that enables the merging of data from heterogeneous biomedical sources based on the semantics of their data elements.

A mapping of the biomedical data elements (clinical cases) from different, but related, datasets into categories and merged sub-categories to design an integrated database schema.

The remainder of the paper is organized as follows: Section 2 presents related work; Section 3 discusses our system implementation and design choices; Section 4 describes evaluation results and Section 5 discusses the evaluation methodology. Section 6 provides discussion about the problems in healthcare data integration and our solution toward these problems. Section 7 discusses our research work conclusions and future directions.

### 1.2. Terminology

#### 1.2.1. Sub-categories

We define sub-categories as the titles already provided by dataset providers to identify the type of information in the case. For example, “Diagnosis” provides information about the patient's illness, “Differential diagnosis” discusses several possible conditions of the patient based on their clinical symptoms.

#### 1.2.2. The semantics of super-category

The semantics of clinical cases provides the information of the clinical cases, by identifying terms from our data sources. Based on our evaluation, we consider semantically similar sub-categories with at least 80% Hausdorff distances within clusters that are between 0 and 10 range.

#### 1.2.3. Category or attribute name

Through our analysis, we are creating categories - which are the merged super-category combinations that can be used as an attribute while designing a database schema.

## 2. Background and related work

Our literature survey primarily focuses on studies about techniques and existing systems for clinical data integration used in hospitals. We also discuss different techniques used by industry in order to reduce data variability in different data sources. Several research studies have investigated the topic of relational database integration. Common data models Microsoft^4^ are used to share data and its meaning across different applications and business processes, but this applies only to relational databases. Limited research work has been reported for unstructured data, especially for clinical data. Many hospitals are using tools that adhere to the Health Level 7 (International, 2018) standard for the exchange of data among different facilities. Fast Healthcare Interoperability Resources (FHIR) HL7FHIR^5^ is a collection of different standards that defines “Resources” representing different clinical concepts. These resources are based on XML or JSON structures, and all resources have a Unified Resource Locator (URL) associated with them. However, these solutions are standards defined to exchange healthcare data and not data integration systems that will aggregate different data sources into a unified repository. Industry approaches seek to avoid mismatch of sub-categories by standardizing data collection in medical research field. For example, Food and Drug Administration enforces clinical experts to use standard formats defined by the Clinical Data Interchange Standards Consortium Study Data Tabulation (CDISC - SDTM) CDISC.^6^ The CDISC-SDTM is a framework that is used to organize clinical data for human and animal studies. Another example is, for COVID-19, the WHO (World Health Organization) proposed a case report form that all researchers should use, so that data could easily be shared among research groups and countries. This requirement enforces that researchers use the same format to report their findings across different institutions. Clinical researchers were able to validate different studies efficiently (in terms of time) due to this standardization. All these techniques are contributing greatly to the data integration process. However, data standardization after the fact remains a major concern observed by the clinical industry; developing a comprehensive approach that can be used by all hospital is a great challenge in itself. Our algorithms try to reduce the variability in data sources without going back to original data sources and incorporate merging sub-categories to create an integrated repository.

Seneviratne et al. (2019) is a great motivation for our work. Specifically, they discuss the importance of data integration in medical data analysis science. The authors present a survey of methods for merging heterogeneous medical data and related technical, semantic, and ethical challenges in integrating these data. The authors argue that the integration of medical data sources significantly improves the performance of prediction algorithms, knowledge discovery, and diagnostic processes. Revesz and Triplet (2010) discuss the reasons why data integration using classifiers is difficult and present an approach for classifying data based on reclassification (classification performed on classifiers). The authors suggest that data integration using classifiers faces several challenges such as missing values and adequately addressing privacy concerns. Data integration process needs raw data which might not be available due to different constraints (e.g., privacy, security, interoperability). Authors used models shared by hospitals, so they do not have to deal with all these data constraints. The authors used data classifier models shared by different hospitals (instead of using raw data) and then integrated classifiers to build a new classifier. Their approach differs from ours since we work with unlabeled datasets which cannot be used with classifiers. Stonebraker and Ilyas (2018) discuss the status of data integration through a software product called Tamr (https://www.tamr.com/). The authors discuss data integration challenges and practical aspects of schema mapping with the classification of data. Their data integration process needs domain experts, which can be prohibitively expensive in practice. So, authors suggest that future data integration systems should be designed in a way to require less human interaction. Research in the domain of data collection and integration has contributed to patient care and adopting artificial intelligence techniques in their tools [European Society of Radiology (ESR), 2019; Paranjape et al., 2020; Orthuber, 2020]. representation toward the integration of large datasets.

Toward improving biomedical data integration techniques, we have previously developed and reported on Integrated Radiology Image Search framework as a pilot for a radiology data source and medical ontology integration system that provides text-based relevance search (Deshpande et al., 2017, 2019a). IRIS is a radiology search engine that supports natural language queries (Deshpande et al., 2018). Further, we designed a data integration and indexing system, a framework that would enable data sharing and search across biomedical data sources (Deshpande et al., 2019b). We also investigated techniques to summarize and find the coverage of medical ontologies over the medical data sources to interpret their contents. We designed a workflow that can be used to clean medical data sources before integration (Deshpande et al., 2020b). Although data cleaning research is a well-established field, data cleaning often requires domain-specific considerations, and the challenges of medical data integration have not received much attention so far. Building on our previous work, in this paper, we propose the data clustering algorithms that categorize and merge heterogeneous biomedical data based on the underlying semantics of the data elements.

## 3. Materials and methods

In this section, we discuss our methodology for grouping/clustering and merging heterogeneous data source sub-categories. We used hierarchical clustering technique and incorporated human feedback to choose thresholds and compute confidence levels in outcomes of clustering and merging data.

### 3.1. Data sources and medical ontologies for integration

We focus on three types of data: a) Radiology teaching files or teaching files used by doctors and radiologists, b) Clinical reports, and c) Research dataset.

#### 3.1.1. Medical teaching file

A radiology teaching files system is a collection of important cases for teaching and clinical follow-up, and references to understand the variety of diseases. Teaching files share a similar overall structure, but significant variations exist even within the same data sources. Teaching files can include information such as patient history, findings, diagnosis, differential diagnosis, discussion, comments, references, and images related to clinical reports. We integrate MIRC RSNA,^7^ and MyPacs (Group, 2017) teaching cases data sources.

#### 3.1.2. Clinical reports

From our survey of different research institute datasets, we learned that most of the clinical report types of data in the healthcare domain are images (e.g., CT, X-ray, MRI). Those images could be stored in JPEG, DICOM, or PNG formats. Text data associated with those images are patient data such as patient age, date of birth, gender, diagnosis, findings, the status of the case (abnormal/normal). Note that not all records have text reports associated with image data, but images without text reports always have metadata associated with them. We use these metadata to categorize images and to help design the data integration approaches. For this step, we integrate data from NIH clinical reports NIH (see text footnote 2) and EURORAD (Neutorgasse, 2017) clinical reports.

#### 3.1.3. Research datasets

These datasets are used by biomedical researchers to predict different diseases and help improve the diagnostic process. For this, we use data from the NIH chest x-ray NIH^8^ dataset. This is a dataset compiled by NIH that provides images from more than 30,000 patients and over 100,000 anonymized chest x-ray images and associated data.

#### 3.1.4. Medical ontologies

Medical ontologies provide definitions, synonyms, and conceptual relation information for medical terms. Our algorithm uses two medical ontologies, RadLex RSNA^9^ and SNOMED CT (SNOMED, 2017). We used medical ontologies to develop coding standards for different medical terms, develop abbreviation dictionary, and to find synonyms for medical terms. Coding standard document included all relevant definitions (e.g., medical term synonyms) and pertinent information about the diseases (for evaluators with no medical training).

### 3.2. System architecture

In this section we discuss system architecture that we designed to cluster sub-categories and merge them for data integration.

**Figure 2:**
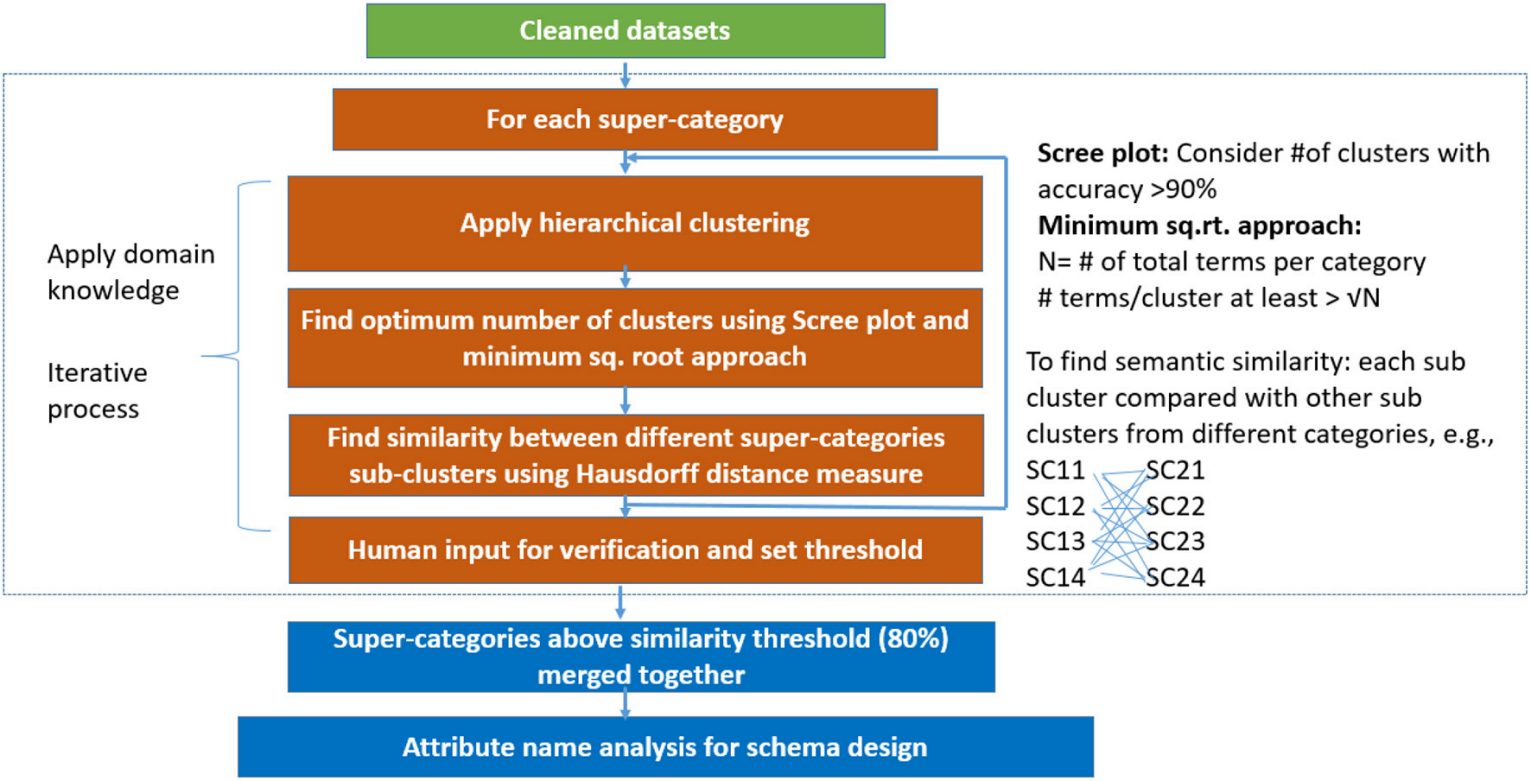
Data categorization and integration system flow (SC, super-category cluster).

As shown in Figure 2, our data categorization work starts by collecting cleaned heterogeneous data sources (based on data cleaning algorithm by Deshpande et al., 2020a). These data cleaning algorithms are used to replace the missing category contents and removal of errors and inconsistent values from different data sources. We choose to replace missing category in a report using another category based on a similarity threshold. To measure the similarity between two categories, we used the Gestalt pattern matching similarity metric Wikipedia.^10^ We also apply stemming, lemmatization (removing inflectional endings—e.g., “studies” and “studying” are converted to “study”) using python NLTK library (https://www.nltk.org/), language identification, garbage characters removal, and removal of stop-words. Stop-words are the most common words used in a language, removed in natural language processing because term frequency of these words would be higher than other important words in corpus (e.g., “the”, “but”, “and”). Using medical ontologies (RadLex and SNOMED CT), we created our own list of stop-words that we did not remove from our data. For example, “with” or “no” are stop-words. However, in the medical domain these terms are significant and may belong to an ontology entry or modify other medical terms. We have identified 24 custom stop-words that we keep in our dataset such as most, between, no, below, or with. After cleaning of the data, we generate term frequency-inverse document frequency (tf-idf) matrix for all terms in the corpus and use this toward forming clusters of these clinical data. For example, for MIRC findings super-category we had total number of cases 2,319 (rows) and total terms were 5,500 (columns). We used hierarchical clustering algorithm with Ward's linkage distance (Murtagh and Legendre, 2014), which minimizes the total within-cluster variance. We use Euclidean distance to measure similarity between documents and Ward's linkage distance to measure similarity between clusters of reports.

We categorize data based on the semantics of the data elements. We identify terms from our data sources that provide semantic information of the data elements. We select different sub-categories and apply hierarchical clustering with different criteria to select the appropriate number of clusters (see Section 3.3). We used ward-linkage distance (Murtagh and Legendre, 2014) to compare these clinical cases from our datasets. This distance is used to minimize the total within-cluster variance. In the next step, we find the semantic similarity between super-category sub-clusters. We leverage human feedback to set a threshold of similarity between different super-category sub-clusters and to evaluate data categorization outcomes. We merge the sub-categories with semantic similarity and then we created a new attribute name for our integrated schema based on the contents of the merged sub-categories. We iteratively perform data categorization for any newly added data sources and expand the schema model, as necessary. Our algorithm is designed in a way that, to add any new super-category, we can take a sample subset of merged categories and our repeat steps of data categorization.

### 3.3. Categorization of medical data elements

To categorize the data, we look for semantic similarity between data source sub-categories. We seek to find the semantics from subgroups, which we formed by dividing super-category into clusters. Number of subgroups equal to number of clusters for respective super-category.

We used hierarchical clustering to form groups of cases for each super-category. We performed clustering on all sub-categories (e.g., history, findings, diagnosis) of medical datasets (e.g., MIRC, EURORAD). We chose hierarchical clustering because it works well with structures and substructures (nested partitions), which we expect in our clinical datasets. Also, we can find arbitrary shape clusters using hierarchical clustering.

We use biomedical data sources with specific focus on medical reports where the data is represented in terms of patient diagnosis, findings, differential diagnosis, history etc. These data come with special properties with many related relations between concepts so when trying to discover themes or categories, partitioning the data is not an ideal solution. Clustering is the process of finding natural groups within a data set such that patterns within a group are more similar to each other than patterns belonging to different groups. Clustering is a difficult problem with complex mathematical modeling. There are two major types of approaches, fast, non-deterministic partitioning techniques, and slower finer-grained hierarchical techniques in addition to more specialized methods (e.g., density based and grid-based clustering (Amini et al., 2011). These approaches differ considerably in terms of efficiency, cost, solution quality, etc. Each approach has its strengths, weaknesses, and limitations. Clustering has been used in a wide range of scientific and engineering applications. In practice, the “best” method is the one that produces the most interpretable results as there is no universal optimum way to select the number of clusters (Rosenberger and Chehdi, 2000). Due to the interrelated nature of our data, we do not expect distinct convex shape clusters but related structures and sub-structures, hence we use hierarchical clustering to discover categories in our biomedical data. We used Euclidean distance after experimenting with different measures like Manhattan and Cosine distances. Our experiments were focused on looking at top frequent terms from theses clusters (with different distance measures) and counting the number of terms which were correlated to each other. We used a multi-annotator consensus of two evaluators to assess the quality of the clusters and determine that Euclidean distance retrieves more meaningful clusters. For example, the top frequent terms from clusters for one meta-category would be anomaly, congenital abnormality, atresia, defect with the Euclidean distance; multifocal, adrenal cortical carcinoma, extra-adrenal, eaten, umbilical artery with the Cosine distance; and shunt catheter, parinaud, concomitant, abutment, nonproductive with the Manhattan distance.

**Figure 3:**
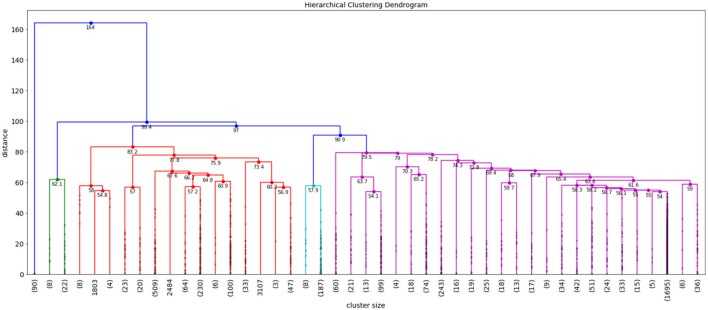
Example of dendrogram for NIH clinical reports indication super-category.

**Figure 4:**
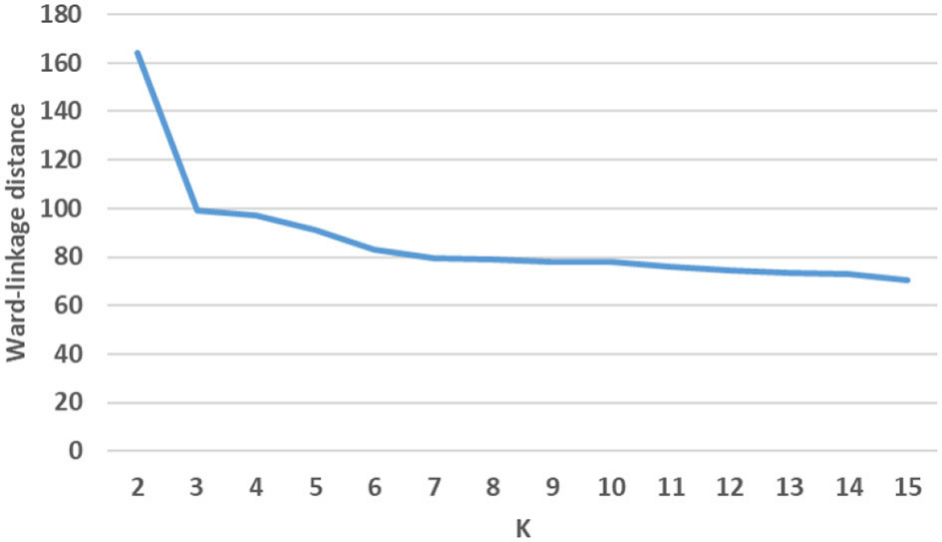
Example of distance plot using ward-linkage.

We used Hierarchical Agglomerative Clustering (HAC) to group objects in clusters based on their similarity. HAC does not need to know the value of k in advance, partitioning data involves multiple steps that assigns number data points to number of clusters and then merge that most similar clusters based on the similarity (distance between each of the clusters), until we get a single cluster. Therefore, to refine the number of clusters we used HAC with intermediate steps, which worked well for our datasets. We considered values of k (number of clusters) between 2 and 150 and used classification and regression tree (CART) (Crawford, 1989) classifier to determine the best initial number of clusters where the classes were the k clusters, the splitting criterion was entropy, and minimum number of samples per node was varied to avoid overfitting. We selected the minimum value of k for which there was a significant decrease in the performance of the classifier when the number of clusters was increased to k+1. This process resulted in grouping each repository into 45 clusters—we then further clustered these 45 clusters into fewer clusters (making it easier to interpret) using HAC with Ward's linkage distance (Murtagh and Legendre, 2014), which minimizes the total within-cluster variance. Our intermediate steps include the following. First, to select the initial number of clusters, we use a scree plot to graph classification membership accuracy of relevant cases. Cluster analysis accuracy is verified using scree plot accuracy. The scree plot shows that our cluster membership classification accuracy is above 90%. Clusters with data membership classification accuracy greater than 90% are used as the initial number of clusters. We use the minimum square root measure to refine these clusters. In this approach, our criteria is to chose the number of clusters where the minimum number of terms per cluster is greater than $\sqrt{N}$, where N is the number of unique terms for that super-category. Using the minimum square root approach, we get two different values of “k.” For example, the “Indication” super-category from the NIH clinical dataset, 1,404 unique terms and as per this criteria minimum unique terms per cluster should be 37. For the “Indication” super-category our clusters fulfill minimum square root approach at the minimum square root approach at *k* = 2, however from 2 to 12 clusters, all clusters are larger than $\sqrt{N}$. For this criterion, we chose the maximum number of clusters to perform a detailed analysis of each super-category. To confirm the number of clusters, we also find the distance within-clusters using a dendrogram. Furthermore, we use dendrogram (as shown in Figure 3) to visualize the hierarchical clustering results and a distance plot to visualize Ward-linkage distance within clusters (as shown in Figure 4). We compare the clusters and use these distances to find the optimal number of clusters with distance plot of Ward-linkage. We analyzed the dendrogram and observed that we can choose *k* = 3 (where a knee in the plot is present); however, we also noted that there is a small distance difference from *k* = 6 to *k* = 15. Short vertical distances indicate close similarity between clusters. We could choose the maximum, *k* = 15 or using the minimum square root guideline, *k* = 12. Based on agreement between these two methods, we decided final number of clusters (in this case *k* = 12) for an individual super-category (results are discussed in Section 4). In cases where there is no agreement between these methods, we can choose the maximum number of clusters.

### 3.4. Super-category merge combination analysis

The categorization of super-category into clusters provides the semantics (using terms and their appearance in clinical cases) behind different data elements. Next, we merge semantically similar sub-categories to design the schema for medical data sources. We calculate the similarity among clusters of different sub-categories and choose a confidence level (a threshold based on experimental evaluation) to match sub-categories from different data sources. We used the Hausdorff distance (Wikipedia, 2020) to measure the distance between super-category clusters. As shown in Equation (1), we calculate distance between clusters from 2 sub-categories (A and B). *d*_*H*_ is the maximum distance between two non-empty sets of A and B. In this equation *sup* represents the supremum (least upper bound) and *inf* the infimum (greatest lower bound).


(1) $$\begin{array}{l} d_{H}\left(A,B\right)=\max\left\{\underset{a\in A}{\sup}\underset{b\in B}{\inf}d\left(a,b\right),\underset{b\in B}{\sup}\underset{a\in A}{\inf}d\left(a,b\right)\right\} \end{array}$$

We compute the distances between sub-categories by calculating the distance from each cluster of one super-category to all other clusters from other super-category. We partition the distance range into 0–10, 11–20, 21–30, 31–40, and 41-above buckets—we have this range of distance based on our analysis that shows that data lies within maximum distance of 50 (no data above distance 50). Our evaluation shows that smaller distance measure corresponds to higher similarity between clusters from sub-categories. Clusters with distances that fall within the first range of 0–10 are semantically similar to each other (results discussed in Section 4). Super-category sub-clusters with distances that fall in the second range of 11–20 often provide semantically similar elements, but some of the cases are not similar. It is unlikely that all clusters from sub-categories will have distances range between 0 and 10. So, to decide threshold to merge sub-categories along with manual evaluation we used heuristic measurement, in which we picked 10 sample sub-categories to check how many sub-categories fall in the range of 0–10 distance. Our analysis shows that we can merge sub-categories in which a minimum of 80% distances within clusters should lie between the 0–10 range. We also find that the distance directly within sub-categories (without clustering sub-categories) and our analysis shows that we lose important information (semantics) if we directly measure distances within full sub-categories (results discussed in Section 4). We calculate similarity between each cluster of one super-category with each cluster from other super-category (many-to-many—as shown in Figure 2) and set a threshold to match sub-categories. If the sub-categories clusters similarity (Hausdorff distance) is above the chosen threshold then we merge that super-category combination into a new attribute. All pairs of super-category combinations with similarity score exceeding a given threshold are returned as matches and define the database schema. Images are categorized based on meta-data or text associated with those images. In some of the data sources, there is meta-data associated with images and no text reports (e.g., NIH chest-x-ray image datasets with diagnosis information). We consider this information as a super-category to merge these cases with other sub-categories, using the same algorithm as for regular sub-categories.

### 3.5. Evaluation of super-category merge combination

In this section, we discuss our evaluation criteria for super-category clustering results and merging of super-category combinations. For evaluation purposes, we used the coding standards that we designed using medical ontologies [RadLex RSNA^11^ and SNOMED CT (SNOMED, 2017)] that explain ontology terms synonyms and definitions. Our evaluation was focused on observing contents of sub-categories and look for overlap of most commonly used ontology terms and appearance in different sub-categories. For example, left, right position of body part, imaging sign, and margin (angular or irregular margin) are all ontology terms that represent observation of a particular clinical case. So, if the term appears as the super-category then these contents belong to observation category.

#### 3.5.1. Analysis of top-frequent terms

We evaluated top frequent terms from different super-category combinations, where merge analysis matches our distance threshold approach (sub-categories at are merged based on Hausdorff distance). We look for frequent terms and interpret relevance of terms using our coding standards and then we evaluate merge super-category combinations. If we find frequent terms (minimum 2) relevant to super-category and their appearance is logical with ontology entities or title of the sub-categories then we consider that is a correct combination.

#### 3.5.2. Evaluate sample number of cases

We manually evaluated 20 (randomly chosen) cases from the merged sub-categories, which are merged based on our algorithm. Our evaluation is binary (YES/NO), based on relevance of the cases as it relates to the title of merged combinations. We find relevance using our coding standards and documentation that provides details about clinical terms, systems, and corresponding definitions. From these cases, we look for the contents and ontology terms that provide information about that case. While manually evaluating these cases we also look at the overlap of top most frequent terms. Evaluators use this document to find the relevancy between frequent terms and clinical case contents. We evaluated total 10 super-category combinations with 20 sample cases for each combination. Our analysis shows that our relevance score for these results was more than 80%. When we looked at the false positive cases, we observed that we have cases where contents of different sub-categories have some overlap of terms from different sub-categories, which is not surprising as this is the nature of clinical data. For example, case with diagnosis of renal failure also discuss history of diabetic in the discussion. In this example, terms related to history super-category also appears in discussion super-category.

#### 3.5.3. Distances within full sub-categories

To evaluate our clustering approach, we also compute distances directly between 10 sample sub-categories before we classify them into clusters. Our analysis shows that direct distance within super-category is not as effective for finding semantics behind clinical cases, as these results shows many of the dissimilar cases; more than 60% of Hausdorff distances within sub-categories are above 30, which ultimately not useful to merge similar sub-categories.

Our evaluation analysis and results are discussed Section 4.

## 4. Results

### 4.1. Clustering with semantics of data elements

**Figure 5:**
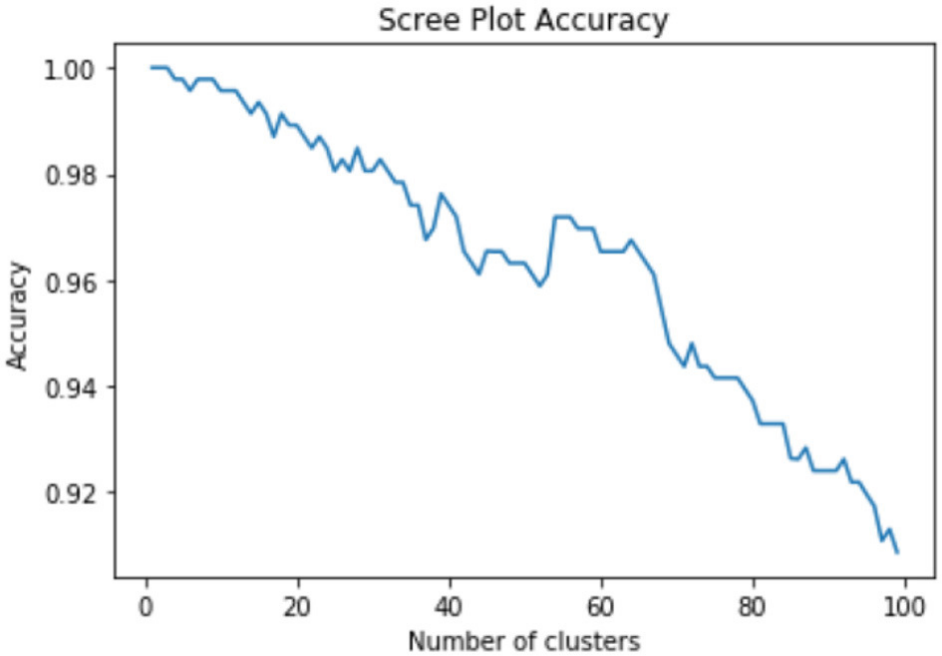
Cluster membership accuracy scree plot.

As our datasets are not labeled, we rely on unsupervised machine learning techniques to categorize data elements. We used HAC with Ward's linkage distance (Murtagh and Legendre, 2014) which minimizes the total within-cluster variance, in order to cluster sub-categories. We used a scree plot to visualize membership classification accuracy of cases, which is used for categorization of data elements of different number of sub-clusters for sub-categories. From the scree plot in Figure 5. We can observe that for all clusters we get high classification accuracy with respect to the number of clusters (approximately 96% of clinical cases are correctly classified into their chosen clusters at this point). Further, and because it is the minimum value of k (which is 100) for which there is a decrease in the performance of the CART classifiers for NIH clinical cases. We also used scree plot to evaluate the classification accuracy for 10 different sub-categories (chosen based on super-category captions that do not share similar captions (not having same captions) and observed that we get a minimum 90% accuracy with 100 clusters or more.

**Figure 6:**
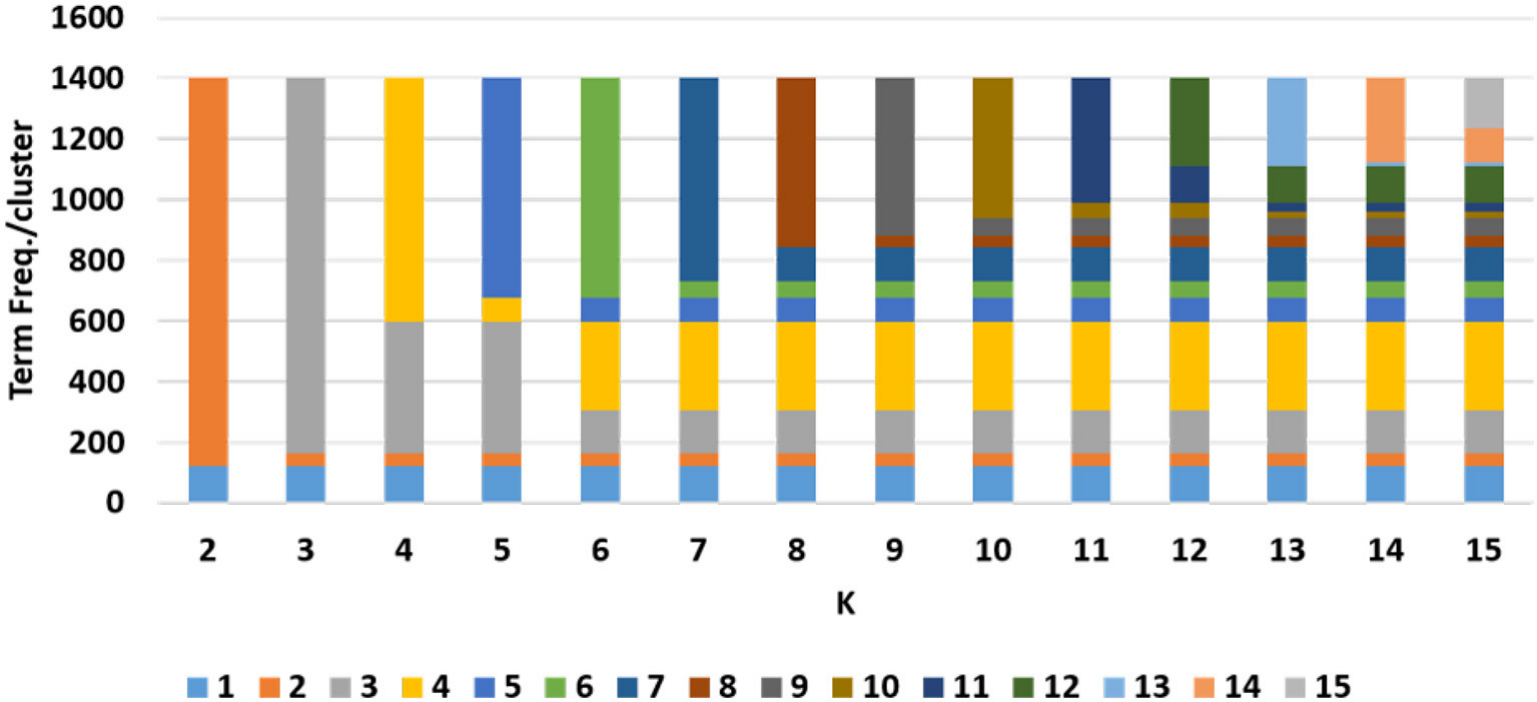
Example of term frequency analysis to decide number of clusters.

So, we decided to choose value of k with classification accuracy greater than 90%. Looking at scree plot of accuracy is not enough because analyzing at least 100 clusters that are above the 90% accuracy threshold will require a lot of human labor. Further, we also wanted to capture context of the data elements. Therefore, as discussed in methodology Section 3.3, we use an additional technique that choose the clusters using the minimum square root approach. This helped us determine the number of clusters based on minimum unique terms per cluster. As shown in Figure 6, we performed analysis which evaluates clusters based on the number of terms present in individual clusters, and identified the value of k where clusters will have fewer than $\sqrt{N}$ terms, when we stop further clustering. Figure 6 shows an example of clustering for NIH clinical reports indication category. The category has 1,404 unique terms ($\sqrt{1,404}$ threshold is 37). After *k* = 13 we get clusters with values with fewer than 37 terms in each cluster. Therefore, we stop at 12*th* clusters. However, one can still consider between 2 and 12 clusters for this example based on the minimum square root approach. Further, we used dendrogram to visualize the hierarchical clustering results (as shown in Figure 3) and a distance (ward's linkage) graph (as shown in Figure 4) in order to validate our minimum square root approach. We find the distances within clusters and use these distance to find number of clusters with distance plot of ward-linkage. We observe from the ward-linkage graph that we can choose clusters at *k* = 3; however, we can also see that there is a small differences within distances of *k* = 6 to *k* = 15. Clusters with less distances are the clusters that representing similar objects (based on our manual evaluation), so we decided to choose clusters within the range of *k* = 6 to *k* = 15. As we wanted to do in depth analysis of clusters and based on our minimum square root approach we choose maximum i.e. *k* = 12 number of clusters. This way we find number of clusters to extract semantic information from individual super-category.

### 4.2. Hausdorff distance for sub-categories similarity analysis

**Figure 7:**
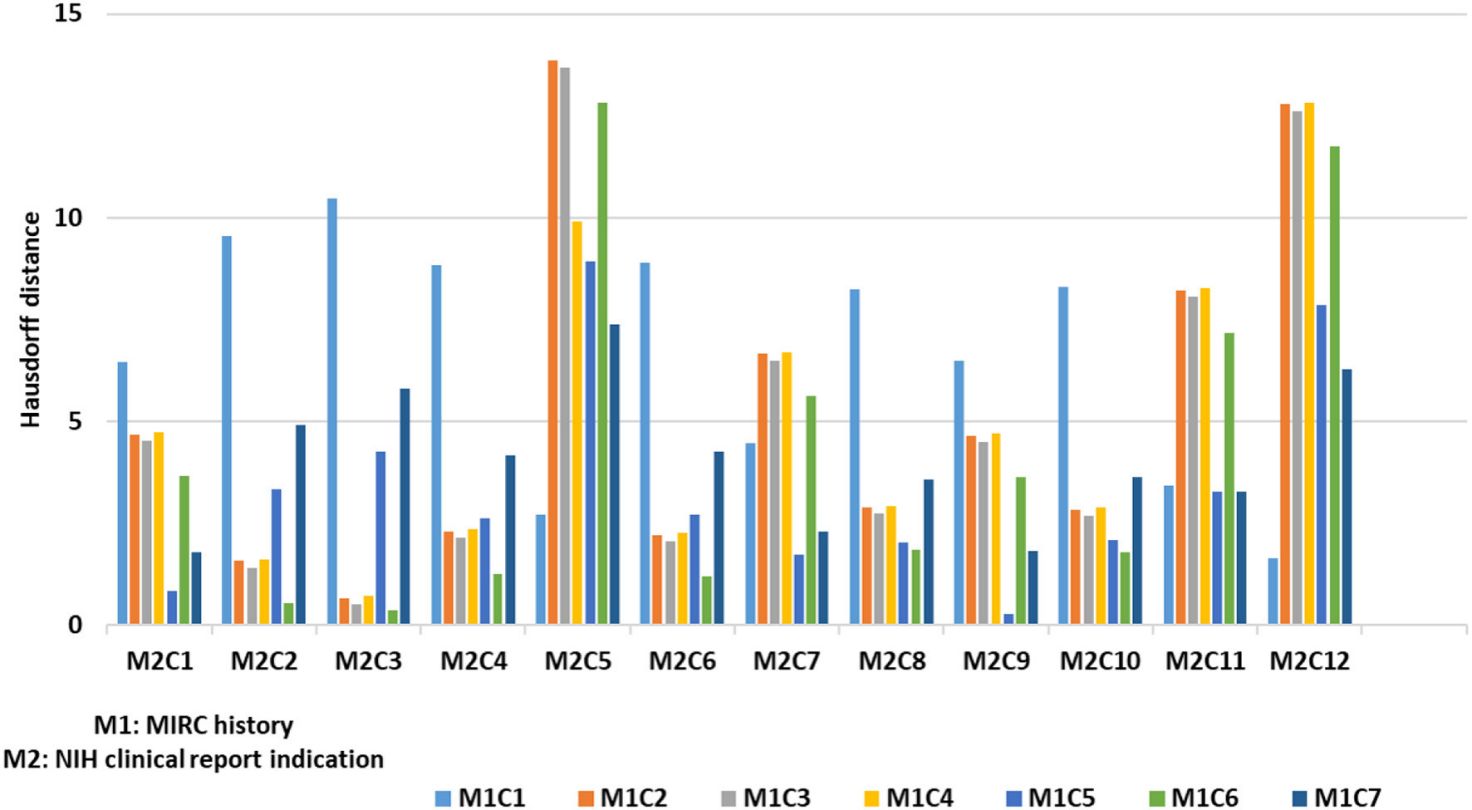
Example of Hausdorff distance between sub-categories clusters.

**Figure 8:**
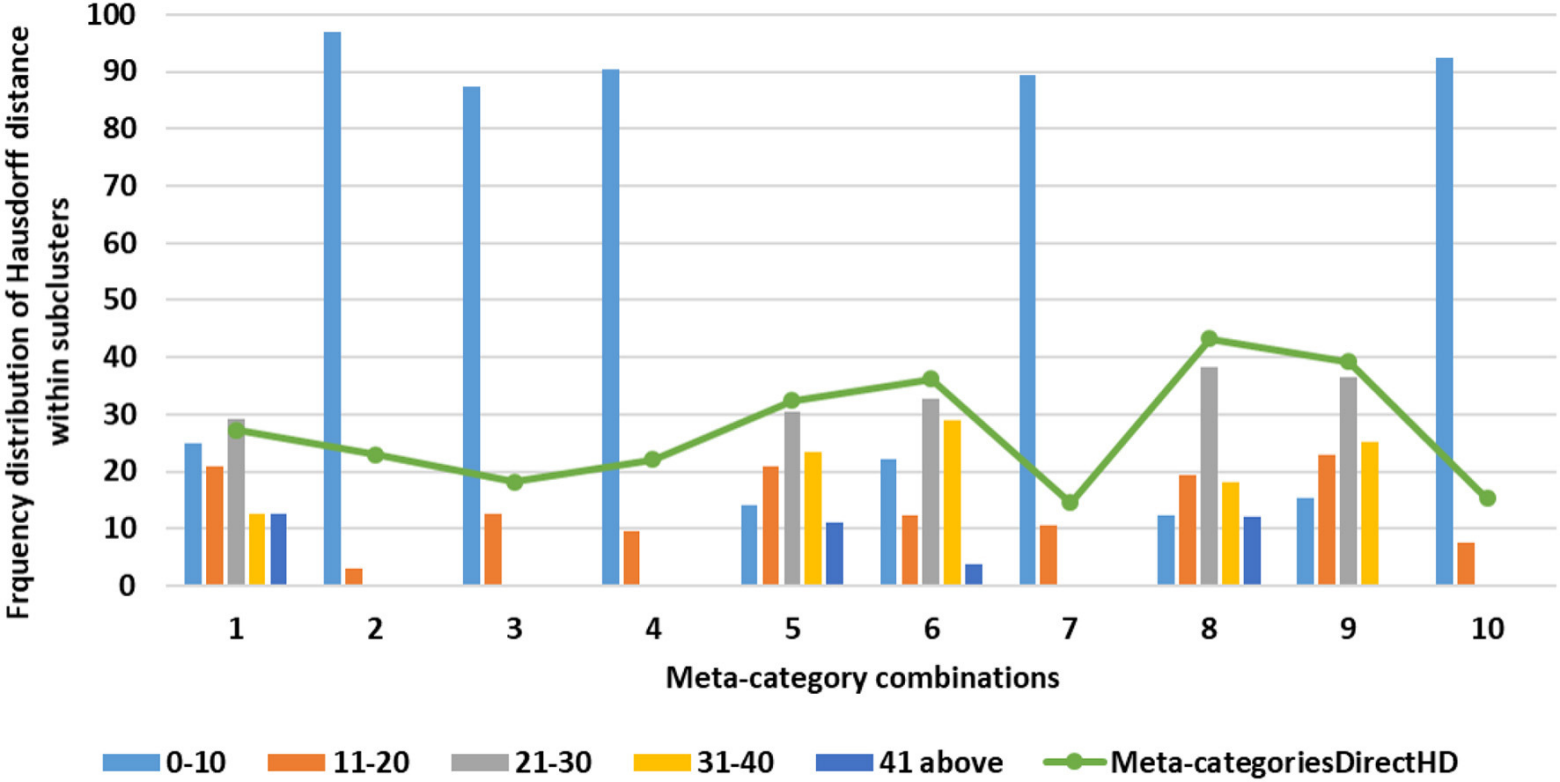
Super-category combination threshold analysis.

Once we categorize data elements from super-category into clusters, our next goal is to detect similarity between pairs of sub-categories; when two sub-categories are similar, we aim to merge these categories into one. As discussed in Section 3, we used the Hausdorff distance to calculate similarity between two sub-categories. We illustrate this process by discussing an example of finding distance between MIRC history and NIH clinical reports indication super-category. For MIRC history category, we have 7 clusters and in NIH clinical reports indication category we have 12 clusters (using our clustering technique that we discussed in Section 3). We calculated distance from each cluster for an individual category to another category (many-to-many). As shown in Figure 7, we obtained 84 distances within these 2 sub-categories with 90% of the distances within 0–10 range—which is within our threshold (as discussed in Section 3.4) and can lead us to merge these categories. We performed this analysis on all super-category combinations. To determine merge criteria for sub-categories we performed a further analysis of finding the distance distribution across all super-category combinations in order to identify the threshold to determine which sub-categories can be merged. As shown in Figure 8, we calculated frequency distribution of Hausdorff distance within all clusters from different super-category combinations. We partition our distances into ranges of 0–10, 11–20, 21–30, 31–40, and 41-above. We illustrate our analysis using 10 sample super-category combinations. These categories were chosen based on super-category captions—we focused on names that are not obviously similar at a glance (e.g., MIRC history and NIH indication): #1: MIRCdiagnosis _NIHindication, #2: MIRCdiagnosis _MyPacsdiagnosis, #3: MIRCdiagnosis _NIHimpression, #4: MIRChistory _NIHindication, #5: MIRCdiagnosis _EURORADobservation, #6: MIRChistory _EURORADobservation, #7: MIRCfindings _EURORADobservation, #: MIRCdiagnois _EURORADpatient, #9: MIRCfindings _EURORADpatient, #10: MIRChistory_EURORADpatient. From this analysis we can observe that for #2, #3, #4, #7, and #10 super-category combinations, 80% of clusters distances range within 0-10, so we can merge these sub-categories. We also calculated sub-categories direct Hausdorff distance (as shown in Figure 8 with a green line) and, using our heuristic measurement (discussed in Section 3.4), observed that those distances are above 0–10 range and do not provide us with semantically similar data elements. We observed that #7 and #10 super-category combination with direct distances are below 20. Our evaluation shows that those cases (with range of 11–20), do not provide similar cases and further if we observe, our approach provides us 5 merge combinations (out of 10) and direct distance approach provides us 2 merge super-category combination, which ultimately leads us to use super-category clustering approach. We evaluated our super-category combination analysis (as discussed in Section 3.4) and we observed that we can merge sub-categories in which minimum 80% distances within clusters lie between 0 and 10 range.

We have a total of 82 sub-categories and after applying our data categorization and merge technique we were able to merge 72 sub-categories (88%), which resulted in 29 final categories.

**Figure 9:**
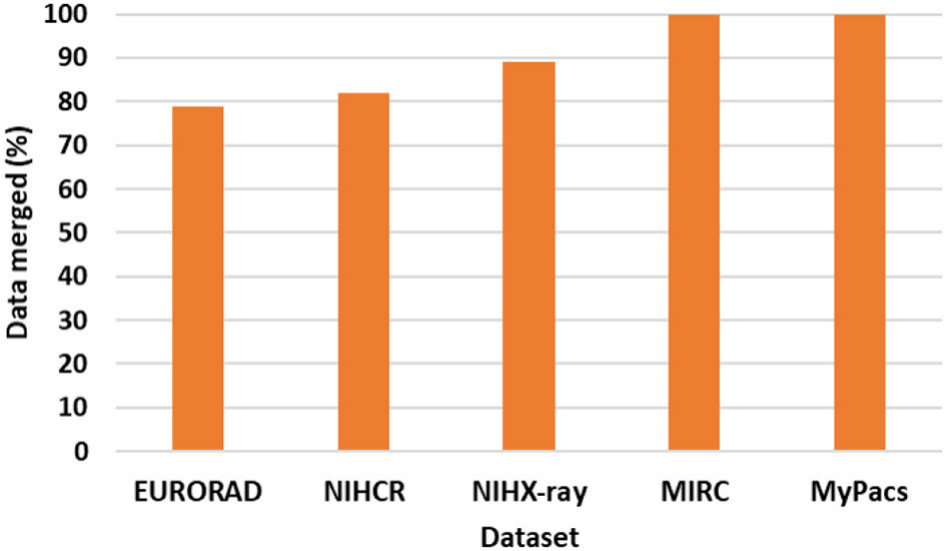
Super-category merge analysis.

As shown in Figure 9 our super-category merge analysis shows that out of 5 datasets, MIRC and MyPacs sub-categories are 100% merged (all sub-categories are merged). EURORAD have total 24 sub-categories and 5 of them did not merge: Area of interest, Imaging technique, Imaging Procedure, Image Origin, and DOI (Digital Object Identifier). From NIH clinical reports 4 out of 22 did not merge: Author affiliation, Case comparison, license type, and license URL and from NIH chest x-ray with 9 sub-categories 1 did not match i.e., Diseases. We were not able to merge 100% sub-categories because we do not have datasets that provide cases with semantically similar data elements and that did not cross our threshold that we use to find distance within different sub-categories. Our next step is to a design a database schema.

### 4.3. Database schema attribute name

After merging semantically similar data elements, our next goal is to design a database schema. In order to assign attribute names to the database schema for the newly merged categories we used the following steps: 1) If more than one super-category has the same name, then use that name as an attribute name. 2) If there is no agreement then: a) Find the top frequent terms (to augment that decision) for individual super-category. b) Find the top frequent terms for merged sub-categories data. c) Compare coverage (overlap of frequent terms) of top frequent terms from merged combination with all individual super-category frequent terms. d) Choose the attribute name from a super-category with maximum coverage of frequent terms. e) If two sub-categories have same coverage of frequent terms then look at the ontology entity name (if present) to choose attribute name with top 5 frequent terms from super-category. If no ontology entity name matches with super-category name then randomly choose any of the name within these sub-categories. For example, MIRC Findings, EURORAD Observation, MyPacs Clinicalfindings, NIHCR MedlineCitationFindings (NIH clinical reports dataset), NIHX-ray FindingLabels (NIH X-ray dataset) have semantic similarity above our threshold (80%), so we merged these sub-categories. In order to decide attribute name for these merged data, we apply above steps and find top frequent terms and their overlap with merged data. In this example we get MIRC with highest coverage of merged category frequent terms, which makes merged attribute name Findings.

## 5. Evaluation analysis

### 5.1. Merge data category analysis

To evaluate merge data category analysis results we used 10 sub-categories—as discussed in Section 4.2 (e.g., MIRCdiagnosis-NIHimpression, MIRChistory-NIHindication, MIRCdiagnosis-EURORADobservation). Here we discuss MIRC findings and EURORAD observation sample combinations of sub-categories, because these two sub-categories are examples of having completely different titles—which are representative of our other super-category experiments. As we could assume some obvious merges such as MIRC diagnosis and MyPacs diagnosis sub-categories, we used other combinations such as MIRC finding, EURORAD observation for validation of our results. While evaluating these results, we wanted to see the overlap between terms from merged-categories. We find the top 25 frequent terms from MIRC findings and EURORAD observation categories and checked if there was any overlap within top frequent terms. Within these top frequent terms we observed that 16 out of 25 terms match, which is around 65% overlap. We performed the same experiment with other nine super-category combinations and concluded that we can set 50% as a threshold for overlap in term in order to validate merge super-category combinations. We do not ignore super-category combinations below threshold, we keep those sub-categories as a separate while designing a database schema. As discussed in Section 4.2 we calculated Hausdorff distance between pairs of sub-categories (without clustering) for 10 sample sub-categories and observed that some direct super-category distances were in the range of 20–30, which is not in the range of our semantic similarity threshold (0–10), and therefore does not meet our criteria to merge sub-categories. This demonstrates that our clustering approach is better than calculating direct distances within sub-categories for the purposes of merging.

We also performed a third validation by manually evaluating 20 cases from each super-category. We observed that cases from these sub-categories have more than 60% coverage of “findings” entity ontology terms, which shows that these cases discuss findings behind particular case. As a last validation step we calculated coverage of top 25 frequent terms in those sample 20 cases from each dataset super-category and observed that, in MIRC findings super-category average 20% of terms are top-most frequent terms, in EURORAD observation super-category average 24% of terms are top-most frequent terms. Similarly, we calculated coverage of top frequent terms from other super-category combinations, and observed that 15% has a marginal overlap of terms with a sample set of terms from 20 cases. Our evaluation shows that overlap of term above 15% are relevant sub-categories from merging perspective.

From this analysis we show that our approach to merge different sub-categories based on context of data elements provides category combinations which are semantically similar to each other. Our approach can be used along with existing data standards to exchange patient's electronic healthcare data and make it more accessible across different systems.

## 6. Discussion

In this proposed work we addressed three important questions: Finding semantics of data elements from clinical super-categories, merging super-categories, and labeling of the merged-super-categories, for data integration purposes.

### 6.1. Inter-annotator agreement

We evaluated these merged categories from experts in order to know these merging is useful or not. We used multiple annotators (two from the clinical domain and two from the computer science domain with knowledge of medical data). We manually evaluated 20 (randomly chosen) cases from the merged sub-categories, which are merged based on our algorithm. Our evaluation is binary (YES/NO), based on relevance of these cases toward the title of the merged combinations; we find relevance using our coding standards. Using the medical ontologies RadLex and SNOMED CT, we designed our own coding standard, in which we map clinical terms with their synonyms and definitions. Coding standards (as discussed in Section 3.5) documentation describe details about clinical terms, systems, and corresponding definitions which we designed using medical ontologies (RadLex and SNOMED CT). From these cases we look for the contents and ontology terms that provide information about that case. While manually evaluating these cases, we also look at the top most frequent terms. Evaluators use this coding standard document to find the relevancy between frequent terms and clinical case contents. Evaluators provided us feedback as to whether the merged combination cases were relevant or not. Our analysis shows that 88% of the merged combinations provide relevant results. When we reviewed the remaining 12% of the combinations, we observed that the Hausdorff distance within sub-clusters of these super-categories was above 10. Based on this evaluation, we decided final distance range to merge super-categories when it is between 0 and 10 (that is based on irrelevant categories). We then evaluated total 10 sub-category combinations with 20 sample cases for each combination. These 10 sub-categories were chosen based on their captions—that do not share similar captions (not having same captions e.g., diagnosis, history) and observed that we get a minimum 90% classification accuracy with 100 clusters or more, confirming the choice of our Hausdorff distance threshold. This methodology will help researchers understand the content from different super-categories and use similar analysis to merge extract and merge categories for different datasets.

We used hierarchical clustering to capture semantics from clinical cases. We used CART classification (where the clusters represented the classification classes) to learn the best starting point for our clustering analysis. The scree plots in Figure 5 show the classification accuracy for different number of clusters for NIH clinical reports. We chose 45 clusters as it results in a good accuracy with respect to the number of clusters (approximately 95% of teaching files are correctly classified into their chosen clusters at this point) and because it is the minimum value of k for which there is a significant decrease in the performance (in terms of time) of the CART classifiers for data repositories.

Cluster analysis is verified by building a classifier of the cluster labels and checking for a drop accuracy in Figure 5. For finding similarities between different clusters, we used Hausdorff distance, and this analysis was further used for merging of different sub-categories from these clusters. Our super-category merge evaluation shows that we can merge sub-categories with distance between 0-10. While we do not expect 100% merging due to heterogeneity of data, we were able to merge 88% of data from five clinical data sources. Some data was not merged as it did not meet our threshold of similarity. Further, we name these merged-sub-categories (attributes) for designing a database schema, based on the knowledge we gained from data analysis of these attributes. These results show that our algorithm can be used to categorize and merge clinical sub-categories without any annotations or human experts' involvement. These are the different parameters that affect our data categorizations techniques: We used the different parameters that affect our data categorizations techniques; such as, hierarchical clustering algorithm with Ward's linkage distance, minimum square root approach to decide the number of clusters, and Hausdorff distance to compare similarity between different sub-clusters. We experimented our algorithm on two more datasets from the healthcare domain, the NIH X-ray dataset NIH^12^ and CheXpert dataset,^13^ however we are also planning to check our algorithm on other datasets (e.g., claim dataset, pathology dataset) as a part of future work. The datasets that we used in this paper took an average of 4 min to form these clusters, however, our clustering time may vary for other types of datasets or different sizes of datasets. We tuned these parameters by considering variability between clinical data-sources but these can be changed based on the properties of the datasets. Our future work will focus on developing an annotation-based integrated clinical cases repository from a variety of data sources. We also want to validate our approach by integrating a few more data sources (e.g., clinical trial data, claim data, health surveys) and test this algorithm with researchers who face data categorization problems. In the future we are planning to use these annotations and different clustering criteria to refine, compare these clinical cases and merge toward an integrated repository.

## 7. Conclusions

Heterogeneous and unstructured properties of biomedical data make it challenging to categorize and organize into a unified data repository. Our data categorization technique categorizes and merges these medical data elements based on the semantics of clinical cases. Our approach involves human input to set a threshold for finding similarities between different categories of medical data sources and further evaluate results. In this way, we designed database schema and integrate these merged sub-categories. Our next goal is to develop a correlation-aware index for this integrated repository to enhance recall and performance in terms of the time of data retrieval.

## Data availability statement

The datasets presented in this study can be found in online repositories. The names of the repository/repositories and accession number(s) can be found below: http://mirc.rsna.org/query.

## Author contributions

PD conducted R&D into the system design and methods for data categorization and integration of clinical data, conceptualization, and writing—original draft. She performed an extensive literature study for biomedical datasets integration, learn challenges, and need for proposed system. AR guided this methodology. AR, RT, JF, DR, and SA provided feedback on the features of the framework and helping position it with respect to other research. PD and AR: investigation and methodology. AR, JF, DR, and SA: supervision. PD, HT, and MS: validation. AR, RT, JF, MS, HT, DR, and SA: writing—review and editing. All authors contributed to manuscript writing and editing.

## Footnotes

^1^ http://mirc.rsna.org/query

^2^ https://nihcc.app.box.com/v/ChestXray-NIHCC/folder/36938765345

^3^ https://www.nih.gov/news-events/news-releases/

^4^ https://docs.microsoft.com/en-us/common-data-model/

^5^ https://www.hl7.org/fhir/index.html

^6^ https://www.cdisc.org/standards/foundational/sdtm

^7^ http://mirc.rsna.org/query

^8^ https://www.nih.gov/news-events/news-releases/

^9^ http://www.radlex.org/

^10^ https://en.wikipedia.org/wiki/Gestalt_Pattern_Matching

^11^ http://www.radlex.org/

^12^ https://www.nih.gov/news-events/news-releases/

^13^ https://stanfordmlgroup.github.io/competitions/chexpert/
